# “They’re quite talented”: Nursing Assistant Scope of Practice in VHA Community Living Centers

**DOI:** 10.1093/geroni/igaf122.1156

**Published:** 2025-12-31

**Authors:** Katherine Kennedy, Nathalie Mcintosh, Sylvia Haigh, David Mohr, Hyeyoung Park, Eleanor McConnell, Whitney Mills

**Affiliations:** Providence VA Healthcare System, Providence, Rhode Island, United States; THRIVE, Providence VA Medical Center, Providence, Rhode Island, United States; VA Providence Healthcare System, Providence, Rhode Island, United States; Veterans Health Administration, Mason, Ohio, United States; University of Massachusetts Amherst, Amherst, Massachusetts, United States; Durham VA Healthcare System, Durham, North Carolina, United States; Brown University, Providence, Rhode Island, United States

## Abstract

The U.S. Veterans Health Administration (VHA) operates its skilled nursing homes, known as Community Living Centers (CLCs), using different structures to support nursing assistants (NAs) and nurses’ delivery of care to complex patients as compared to community nursing homes. Structures include adequate staffing, safe patient handling programs and equipment access, effective nurse supervision, higher wages with benefits, and the ability to hire and train NAs without certifications but with relevant healthcare experience in response to recruitment/retention challenges. Thus, a pilot study examined NA training aspects within 4 CLCs in the New England region. Seventeen NAs and 13 nurse leaders (i.e., nurse managers, educators, clinical resource nurse, chief nurse) completed individual interviews (July-October 2024), and a team employed qualitative content analysis using a coding frame based in the Paraprofessional Healthcare Institute (PHI) 5 Pillars of Job Quality. Four themes emerged related to NA scope of practice and care quality: (1) NAs learn advanced clinical skills in partnership with nurses (e.g., electrocardiogram, catheter placement and care); (2) Skills acquisition occurs over years of repetition; (3) Skills are context dependent (e.g., hospice, dementia, subacute); and (4) Insufficient standardization, tailored training, and training resources negatively impact care delivery. In VHA CLCs with nursing oversight and sufficient resources, NA scope of practice appears much greater than in community nursing homes. Research-practice partnerships to design and test learner-centered education using evidence-based models and technologies could benefit current and new NAs by anticipating skills needed with a new admission or using dementia care scenarios with microlearning.

